# Analytical Sensitivity of Lateral Flow Devices against SARS-CoV-2 Omicron Subvariants BA.4, BA.5, and BA.2.75

**DOI:** 10.1128/jcm.01097-22

**Published:** 2022-10-31

**Authors:** Charlene Mackenzie, Mitchell Batty, Georgina Papadakis, Laura Stevens, Yano Yoga, George Taiaroa, Helen Stefanatos, Ivana Savic, Thomas Tran, Joshua Deerain, Jacqueline Prestedge, Julian Druce, Leon Caly, Deborah A. Williamson

**Affiliations:** a Victorian Infectious Diseases Reference Laboratory, Royal Melbourne Hospital at The Peter Doherty Institute for Infection and Immunity, Victoria, Australia; b Department of Infectious Diseases, The University of Melbourne at the Peter Doherty Institute for Infection and Immunity, Victoria, Australia; Cepheid

**Keywords:** antigen tests, COVID-19, SARS-CoV-2

## LETTER

Rapid antigen testing has played a pivotal role in the COVID-19 pandemic, enabling quick identification of individuals with SARS-CoV-2 infection, and rapid institution of clinical and public health measures. In many settings, including Australia, rapid antigen testing is now the main modality of testing, with only one-third of SARS-CoV-2 infections now diagnosed using PCR assays (1). The ongoing emergence of new SARS-CoV-2 variants requires constant vigilance in diagnostic assay performance, including assessments of the analytical and clinical sensitivity of rapid antigen tests. Although amino acid changes in the SARS-CoV-2 spike protein are the characteristic feature of SARS-CoV-2 variants, mutations in the nucleocapsid protein (the diagnostic target of most antigen tests) can occur (1). To date, however, the performance of most antigen tests has been assessed against previously circulating variants, including the Delta and Omicron B.1.1.529 lineages (2, 3). Accordingly, we undertook a rapid assessment of the analytical sensitivity of six antigen tests, commonly used in our setting, against the Omicron sublineages BA.4, BA.5, and BA.2.75.

Representative Delta, BA.4, BA.5, and BA.2.75 isolates were obtained from clinical samples referred to the Victorian Infectious Diseases Reference Laboratory (VIDRL) in Melbourne, Australia. We included a Delta isolate to enable comparison with our previous analytical assessments of antigen kit performance (3, 4). SARS-CoV-2 genomic sequencing of virus isolates was performed following cell culture using the Oxford Nanopore Rapid Barcoding (SQK-RBK110.96) and Midnight RT PCR Expansion (MRT001; both Nanopore technologies) kits. SARS-CoV-2 consensus sequences were generated using the ARTIC Sequencing report generated by the inbuilt Nextflow wf-artic supplied by Oxford Nanopore on their GridION instrument. Lineage designation was assigned using both Nextclade and Pangolin v3.1.16. Isolates were grown as previously described (3) and harvested when cytopathic effect (CPE) was observed. For each variant, we constructed a 10-fold dilution range of quantified virus spanning ~2 × 10^8^ to 2 × 10^5^ copies/mL, corresponding to N gene cycle threshold (Ct) values of ~19 to ~29 on an in-house real-time RT-PCR assay (5), and using droplet digital PCR (ddPCR) (3). Testing was performed in quadruplicate using live virus at Biosafety Level 3, and limit of detection (LOD) was defined as the last dilution where all four replicates were positive. Additional testing was performed on three of the kits most commonly used for testing in our setting. Twenty BA.5 and five BA.4 clinical isolates were selected with Ct values 20.1 to 29.0. BA.5 was selected as this is the current dominant variant in Victoria. Sample volumes applied to the test cassette and incubation time were followed as per manufacturer’s instructions. Results interpretation was performed by two readers as previously described (3). A third reader was used to resolve discordant results.

**FIG 1:**
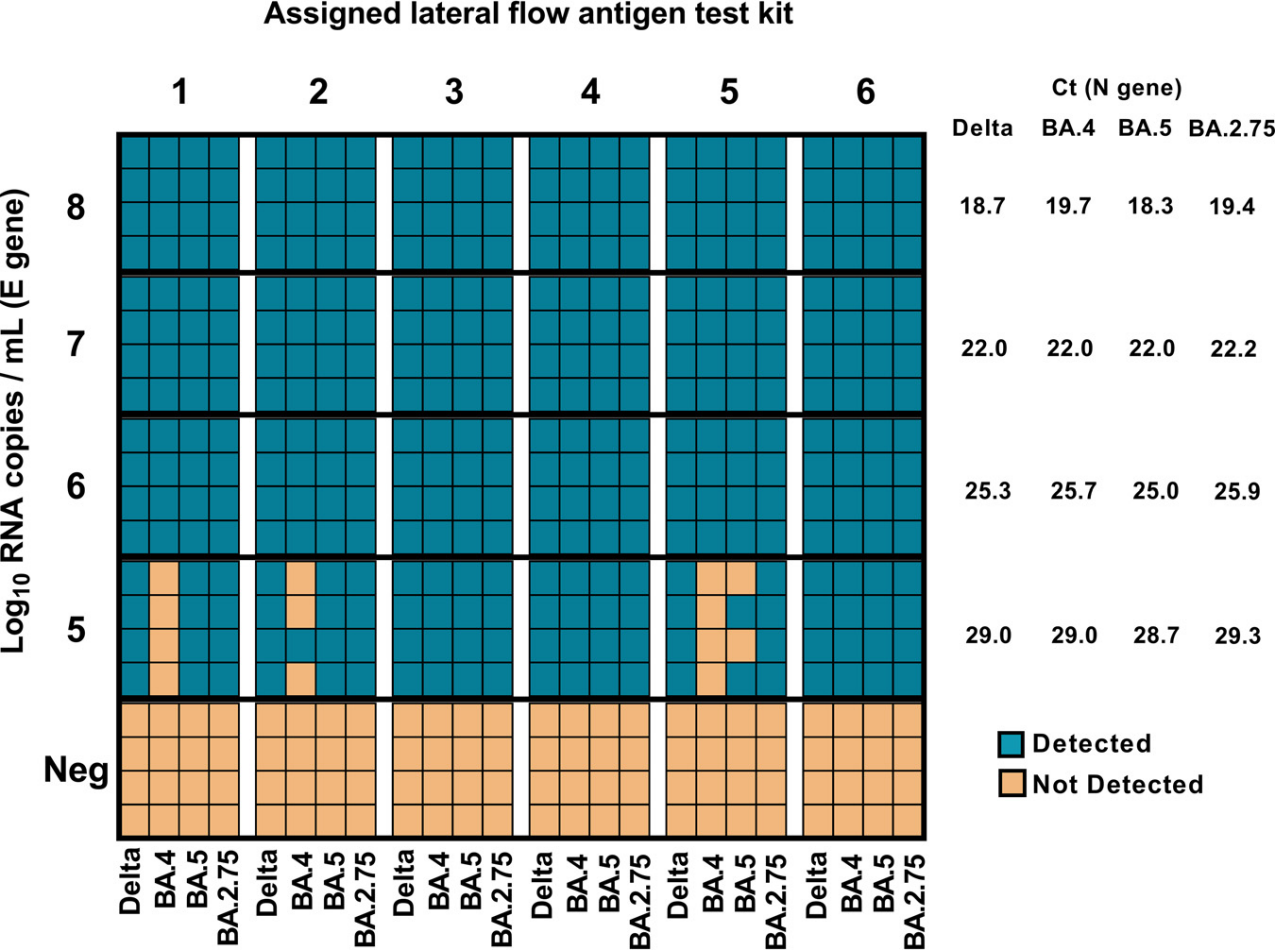
Analytical sensitivities of 6 lateral-flow rapid antigen tests against SARS-CoV-2 Delta and Omicron BA.4, BA.5, and BA.2.75 variants. Antigen kits were tested against 10-fold dilutions (1:10 to 1:10,000) of each variant in quadruplicate. Negative-control samples (Neg) were also tested. Blue boxes signify a positive detection of SARS-CoV-2 antigen in a single replicate, and orange indicates a negative result. Mean cycle threshold values (Ct) for each variant were calculated from triplicate real-time RT-PCR assays targeting the SARS-CoV-2 nucleocapsid (N) gene, with viral RNA copies/mL quantified by droplet digital PCR assays for the envelope (E) gene. The registered names and manufacturers for the antigen tests were as follows: (i) LYHER novel coronavirus (COVID-19) antigen test kit (colloidal gold) Self-Test, Hangzhou Laihe Biotech Co. Ltd. (China); (ii) Testsealabs COVID-19 Antigen Test Cassette, Hangzhou Testsea Biotechnology Co. Ltd. (China); (iii) Panbio COVID-19 Ag rapid test device (nasal), Abbott Rapid Diagnostics Jena GmbH (Germany); (iv) Rapid SARS-CoV-2 Antigen Test Card, MP Biomedicals Asia Pacific Pty Ltd. (Singapore); (v) InnoScreen COVID-19 antigen rapid test device, Innovation Scientific Pty. Ltd. (Australia); and (vi) Roche SARS-CoV-2/Influenza A and Influenza B rapid antigen test, SD Biosensor Inc. (Republic of Korea).

The analytical sensitivity of all kits was similar for Delta, BA.4, BA.5, and BA.2.75 (Fig. 1). All kits detected Delta, BA.4, BA.5, and BA.2.75 at 6 log_10_ copies/mL (Ct 25), and only three kits were able to detect all of the four variants at 5 log_10_ copies/mL (Ct 29) (Fig. 1). Overall clinical test sensitivity was 76% (95% CI 56.2 to 88.8), 88% (95% CI 69.2 to 96.7), and 92% (95% CI 73.9 to 98.9) for Panbio, Testsealabs, and Lyher kits, respectively (Table S1 in the supplemental material), with none of the kits detecting BA.5 above Ct 27.3 (Fig. S1). All kits detected each BA.4 clinical isolate.

Although our data provide information on the *in vitro* ability of antigen tests to detect BA.4, BA.5, and BA.2.75 sublineages at viral burdens similar to those of clinical infections, our results do not directly extrapolate to direct clinical performance. Mean viral burden for clinical SARS-CoV-2 infections can vary between 2 to 8 log_10_ copies/mL depending on specimen type and symptom severity (6–8) and is likely to be impacted by variant and vaccination status. Further, real-world performance of these tests is impacted by many factors, including adequacy of sample collection, viral replication, viral shedding, and tissue tropism. Further clinical monitoring is essential to ensure ongoing adequacy of rapid antigen tests in the face of new variants.
