# Supplementary material for: Analytical Sensitivity of Lateral Flow Devices against SARS-CoV-2 Omicron Subvariants BA.4, BA.5, and BA.2.75
Source: J Clin Microbiol. 2022 Oct 31;60(11):e01097-22. doi: 10.1128/jcm.01097-22 (PMC9667757; doi:10.1128/jcm.01097-22)
Supplement: Supplemental file 1 — Supplemental material. Download jcm.01097-22-s0001.pdf, PDF file, 0.4 MB [file jcm.01097-22-s0001.pdf]

SUPPLEMENTARY RESULTS

Analytical sensitivity of lateral flow devices against SARS-CoV-2 Omicron subvariants BA.4, BA.5 and BA.2.75

Supplementary Table 1. Test sensitivity against a panel of clinical BA.4 and BA.5 clinical isolates. Sensitivity is represented with 95% confidence intervals (95% CI).

	2San Lyher	Testsealabs	Abbott Panbio
**BA.4 isolate**	5/5 (100%, 95%CI 47.8-100)	5/5 (100%, 95%CI 47.8-100)	5/5 (100%, 95%CI 47.8-100)
**BA.5 isolate**	18/20 (90%, 95%CI 68.7-98.4)	17/20 (85%, 95%CI 63.1-95.6)	14/20 (70%, 95%CI 47.9-85.7)
**Overall**	23/25 (92%, 95%CI 73.9-98.9)	22/25 (88%, 95%CI 69.2-96.7)	19/25 (76%, 95%CI 56.2-88.8)

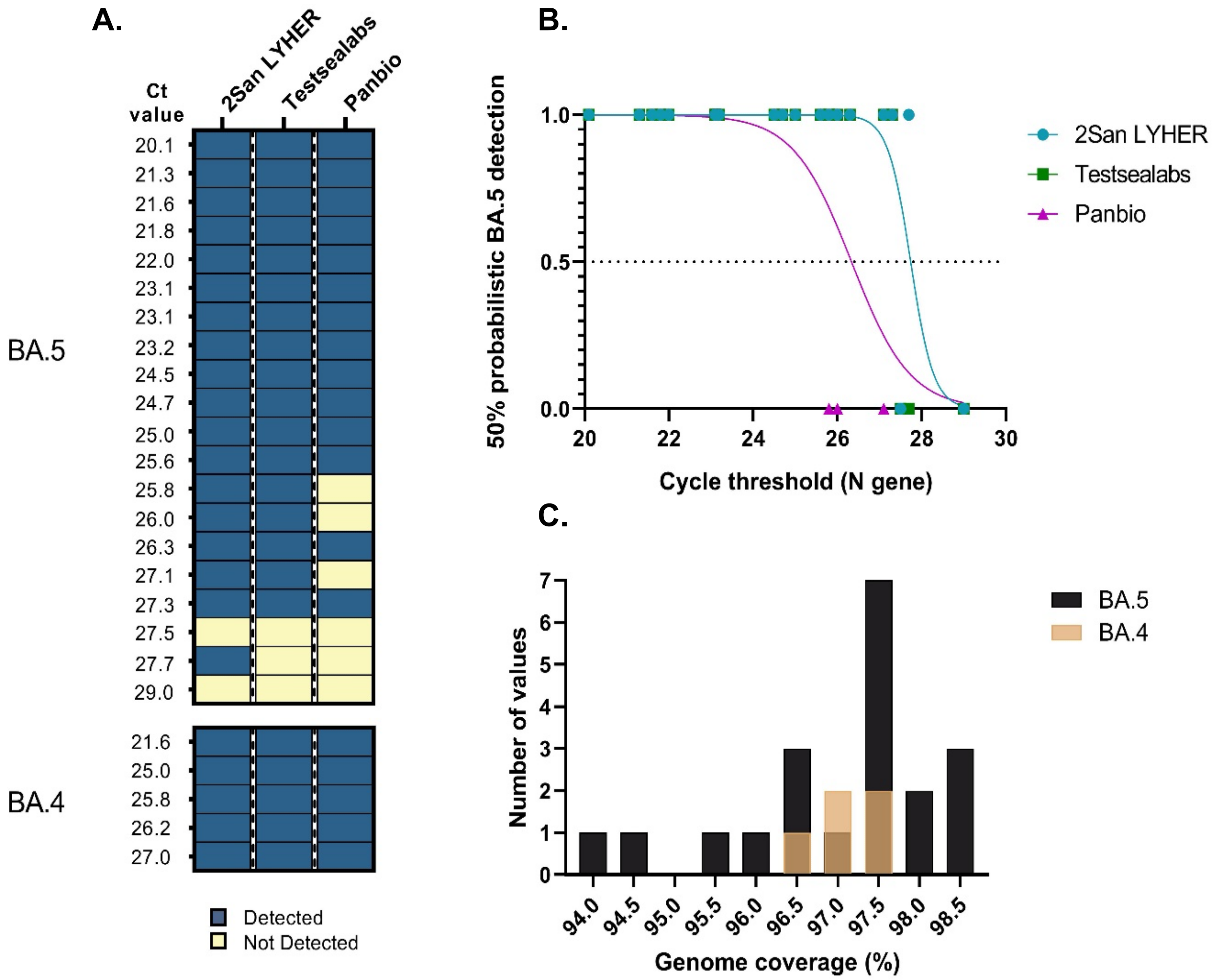

Supplementary Figure 1. Performance of Lyher, Testsealabs and Panbio rapid antigen kits tested against BA.4 and BA.5 clinical isolates. BA.5 isolates (n=20) with an N gene cycle threshold (Ct) range of 20.1 to 29.0 and BA.4 isolates (n=5) ranging from Ct 21.6 to 27 were tested by each kit (A). Logistic regression analysis (B) indicated that the 2San Lyher (teal) and Abbott Panbio (pink) kits had a 50% probability of BA.5 detection at Ct values 27.74 and 26.34 respectively. Testsealabs had perfect separation between positive and negative detection, so a logistics regression curve was unable to be produced. All three kits detected each BA.4 clinical isolate. Genome coverage for each clinical isolate was ≥94.0% (C).
